# STING Agonist VB-85247 Induces Durable Antitumor Immune Responses by Intravesical Administration in a Non–Muscle-Invasive Bladder Cancer

**DOI:** 10.1158/0008-5472.CAN-24-1022

**Published:** 2024-12-19

**Authors:** Miglena G. Prabagar, Michael McQueney, Venu Bommireddy, Rachael Siegel, Gary L. Schieven, Ku Lu, Ruziboy Husanov, Reema Deepak, David Diller, Chia-Yu Huang, Eli Mordechai, Rukiye-Nazan Eraslan

**Affiliations:** Genesis Biotechnology Group, Hamilton, New Jersey.

## Abstract

Bacillus Calmette–Guérin (BCG) is the current standard of care for non–muscle-invasive bladder cancer (NMIBC), but recurrence is common. Additional therapeutic options are a major unmet medical need for treating unresponsive patients. Stimulator of IFN genes (STING) plays a central role in mounting innate and adaptive immune responses to tumor cells, and activation of STING is a promising immunotherapeutic approach. In this study, we developed STING agonist VB-85247 for treating NMIBC by intravesical delivery as a strategy to provide a sustained period of exposure to bladder cancer cells while avoiding potential issues associated with intratumoral injection of STING agonists, which to date have shown only limited clinical efficacy. VB-85247 induced complete response in an orthotopic NMIBC model in contrast to treatment with BCG, which was not efficacious in the model. The efficacious dose was well tolerated and induced an immune response with immunologic memory that protected from rechallenge without further treatment. Activation of the STING pathway via VB-85247 induced upregulation of inflammatory cytokines IFNα/β, TNFα, IL6, and CXCL10, along with maturation and activation of dendritic cells. In addition, VB-85247 provided a therapeutic benefit in combination with immune checkpoint blockade using anti-PD-1 antibody treatment. Together, these preclinical data support the potential utility of VB-85247 for treating BCG-unresponsive patients with NMIBC and for enhancing the clinical benefit of potential of anti-PD-1 in bladder cancer. Based on these data, VB-85247 is being advanced into clinical development.

**Significance:**

STING agonist VB-85247 administered by the intravesical route achieves prolonged tumor regression, induces immunologic memory, and provides additive benefits to anti-PD-1 treatment in non-muscle invasive bladder cancer.

## Introduction

Bladder cancer is a heterogeneous malignancy that often begins in the bladder lining of inner urothelial cells. When tumor cells have not yet grown into the deeper layers of muscle and connective tissues of the bladder, the disease is known as non–muscle-invasive bladder cancer (NMIBC; refs. 1, 2). Bacillus Calmette–Guérin (BCG) therapy is the current standard of care (3). However, about 35% to 50% of patients who initially benefited from BCG therapy develop recurrent tumors within 5 years (4–6). The mechanism of BCG action against bladder cancer is incompletely understood, but IFNs are considered an important part of this process (7). Pembrolizumab, an anti-PD-1 immuno-oncology agent, is an alternative choice for those patients who fail BCG treatment (5, 6). During the KEYNOTE-057 trial, patients who failed BCG therapy received pembrolizumab 200 mg every 3 weeks and showed efficacy with 40% of these patients achieving complete response (8). There is therefore a major unmet medical need for additional therapies.

Stimulator of IFN genes (STING) has recently emerged as a promising target for cancer immunotherapy as its activation has the potential to increase the efficacy of immune checkpoint blockade inhibitors (9–11). The cyclic guanosine adenosine synthase (cGAS)–STING pathway senses DNA in the cytosol. DNA in the cytosol can occur during bacterial or viral infections. Cytosolic DNA can also occur in cancer cells as a result of genomic instability and DNA damage (12). Regardless of the source, cytosolic DNA is a danger signal that triggers innate immune responses. Upon binding cytosolic DNA, cGAS generates the natural STING ligand cyclic guanosine monophosphate–adenosine monophosphate (cGAMP) to activate STING signal transduction. Multiple studies have clearly established a role for STING signaling in innate immune sensing of tumor growth (13–15). Activation of the STING pathway leads to the production of type I IFN, particularly IFNβ, and the subsequent expression of IFN-specific genes such as the chemokine CXCL10 (16). STING also induces expression of proinflammatory cytokines such as TNFα and IL6. The major cellular mechanisms of the STING-mediated anticancer immunity involve type I IFN–induced activation and maturation of dendritic cells (DC), which facilitate cross-presentation of tumor-associated antigens to naïve CD8 T cells in the tumor-draining lymph node and/or in the tumor itself (13, 17, 18).

Studies in mouse syngeneic tumor models confirmed the role of STING signaling in eliciting strong and durable antitumor immune responses (10, 15, 17, 19). Clinical studies of STING agonists to date have primarily utilized intratumoral (i.t.) injection, and until now only limited antitumor activity has been reported (20, 21). The limited activity may be due at least in part to the shorter residence time of STING agonists in the tumor following injection, as rapid absorption from the tumor into systemic circulation has been reported (22). Other factors such as the wide range of tumors examined in the clinical studies with potential nonpermissive tumor microenvironments for T-cell infiltration, poor pharmacokinetic properties, and safety issues may also have contributed to the limited clinical responses (23). We have therefore sought to address some of these issues by focusing on intravesical treatment of a single tumor type, NMIBC. Pharmacologic therapy of NMIBC often utilizes the intravesical route, providing a sustained duration of exposure to tumor cells while limiting systemic exposure. To date, there have been no reports of studies of STING agonists in bladder cancer via intravesical delivery. Therefore, in this study, we have developed a mouse model of NMIBC and utilized the model to evaluate a novel synthetic, rationally designed STING agonist, VB-85247 (Supplementary Fig. S1), via the intravesical route of administration (24, 25). VB-85247 activates STING in mouse and human cells. A single intravesical dose of VB-85247 resulted in activation of the STING pathway target genes such as IFNβ, CXCL10, CCL5, IL6, and TNFα in bladder tissues, as well as maturation and activation of DCs in lymphoid organs. Monotherapy with VB-85247 resulted in up to 100% complete response rate at 40 μg (2 mg/kg) dose level after five weekly treatments. The treatment was well tolerated, eliciting a strong and durable antitumor immune response without any mortality after rechallenge. By contrast, BCG treatment was not efficacious in this model. Whereas anti-PD-1 monotherapy showed only limited efficacy in the NMIBC model, anti-PD-1 combination with VB-85247 resulted in increased efficacy of lower doses of VB-85247. Based on these data, we propose that STING agonist VB-85247 has the potential to markedly improve immuno-oncology strategies that target patients with bladder cancer and extend the clinical benefit of anti-PD-1. VB-85247 is being advanced into clinical development.

## Materials and Methods

### qRT-PCR analysis of STING pathway–targeted gene induction in cells stimulated with VB-85247

#### Cells and reagents

THP1-Dual reporter cells (thpd-nfis, RRID: CVCL_X599), THP1-R232-Dual reporter cells (thpd-r232, PRID: CVCL_A8BI), THP1-Dual KO-STING cells (thpd-kostg, RRID: CVCL_A8AH), and RAW-Dual reporter cells (rawd-ismip, RRID: CVCL_A7ZK) were obtained from Invivogen. Vender-authenticated cells were maintained in culture for up to three passages before compound treatment. *Mycoplasma* contamination was not tested. ADU-S100 (tlrl-nacda2r), Normocin (ant-nr-1), blasticidin (ant-bl-1), Zeocin (ant-zn-1), Qiagen RNeasy Mini Kit (74106), and SuperScript III First-Strand Synthesis Kit (11754-050) were purchased from Invitrogen. TaqMan probes for amplification of human and mouse genes were from Thermo Fisher Scientific (Supplementary Table S1).

#### Compound treatment

Tissue culture cells were seeded at a density of 2 × 10^3^ cells per well in a 6-well plate and stimulated with STING activators at designated concentrations. After treatment, the cells were processed for RNA extraction.

#### Bladder tissues

Whole bladder containing tumor was collected from mice sacrificed at 4 hours, 24 hours, or 6 days after 2 hours of instillation with 40 μg test compound or vehicle. Samples were homogenized and used for RNA isolation.

#### RNA extraction and qRT-PCR

Total RNA was isolated, and cDNA synthesis was carried out on an equal amount of RNA from each bladder or cell preparation according to the manufacturer’s instructions. Target and reference gene expression levels were determined by qRT-PCR using TaqMan gene expression assays and Bio-Rad C1000 Touch Thermal Cycler. Data were normalized to the corresponding GAPDH housekeeping gene, and fold changes in gene induction were calculated by the comparative C_T_ method (26).

### Human primary cells and *in vitro* stimulation with VB-85247

#### Human peripheral blood mononuclear cells

Human whole blood was purchased from Stemcell Technologies (70508) to be used *in vitro*. The blood was diluted at 1:1 ratio with DPBS supplemented with 2% FBS, layered onto Ficoll-Paque Plus in a 50-mL tube, and centrifuged. The cells were resuspended at a density of 3 × 10^6^ cells/mL in media from Cellular Technology Limited (CTLT-010) supplemented with 1× penicillin/streptomycin and 1× GlutaGro from WorldWide Medical Products (61211139) and seeded in 96-well plates. The plates were incubated at 37°C for 16 hours prior to compound treatment.

#### Human primary bladder epithelial cells

Cells were purchased from Applied Biological Materials (T4161) and cultured for 16 to 24 hours according to the manufacturer’s guidelines before compound treatment. Cells with viability greater than 95% were seeded at density of 3 × 10^5^ cells/mL in 100 μL in a 96-well plate.

#### Compound treatment

A half-log serial dilution in endotoxin-free water was prepared for VB-85247 and added to the plates containing cells. The plates were incubated at 37°C. After treatment, the plates were centrifuged at 200 × *g* for 5 minutes, and supernatants were stored at −80°C for protein analysis. The plates containing the cells were stored at −80°C for RNA analysis.

### Mice

Female C57BL/6 mice at 8 to 14 weeks were purchased from The Jackson Laboratory (RRID: IMSR_JAX:000664). Experiments were conducted with 8- to 12-week-old mice in accordance with the regulations of the Association for Assessment and Accreditation of Laboratory Animal Care. The protocol and any amendment involving the care and use of animals in this study were reviewed and approved by the Institutional Animal Care and Use Committee of Genesis Biotechnology Group.

### Bladder tumor model and intravesical treatment

The MB49, a mouse bladder cell line, was purchased from Millipore (SCC148, RRID: CVCL_7076) and transfected in-house with a luciferase-expressing plasmid (MB49-luc) to monitor the growth of orthotopically implanted cells. Before being banked, the cells were tested for *Mycoplasma* by PCR. The cells were passaged twice in DMEM supplemented with 10% FBS and 1× penicillin/streptomycin for inoculation. Prior to instillation of MB49-luc cells into the bladder, mice were anesthetized through inhalation of 1.5% to 3.5% isoflurane while laid on a prewarmed heating pad. A 24-g catheter coated with mineral oil was inserted through the urethra into the bladder, and bladder wall was primed with 0.1 mol/L silver nitrate for 10 seconds to induce a chemical lesion to enable implantation of tumor cells. The content of the bladder was washed out through intraurethral infusion of PBS. Then, each mouse was instilled with a single-cell suspension of 95% viable MB49-luc tumor cells at a density of 0.5 × 10^5^ in a volume of 40 μL. Following instillation, the needle and catheter were left in place for 45 minutes. Treatment of bladder with VB-85247 (in 40 μL at indicated dose levels) or BCG (in PBS at 1 mg/mouse, TICE BCG from Organon Teknika) was done by transurethral instillation through a catheter and allowed to dwell in the bladder for 2 hours. For pharmacodynamics, the animals received a single dose, and for efficacy studies, the treatments were given once every 7 days for 5 weeks. When applicable, anti-mouse PD-1(CD279) antibody (Bio X Cell, BE0146, PRID AB_10949053) was given via i.p. injection twice weekly.

### Serum cytokine and chemokine analyses

Peripheral blood from tumor-bearing mice was collected 4, 24 hours, and 6 days after a single dose of VB-85247 (40 μg, 2 mg/kg) to profile the cytokines and chemokines in serum utilizing electrochemiluminescence multiplex from Meso Scale Discovery.

### Bioluminescent imaging

The growth of intravesically implanted MB49-luc tumor cells was monitored by bioluminescent imaging (BLI) utilizing *In vivo* Imaging System (IVIS; PerkinElmer, RRID: SCR_018621). Mice were injected i.p. with luciferin substrate (PerkinElmer) at 150 mg/kg and anesthetized with 2% to 3% isoflurane, and ventral images of the whole body were taken 15 minutes after injection while mice were placed prone on the heated stage in the imaging chamber of the IVIS system. Following imaging, mice were removed and placed back into their respective cages for recovery. Animals were randomized into treatment groups based on the BLI results to ensure that the tumor size in the bladder was relatively similar in each experimental group prior to treatment.

### Tumor rechallenge and tumor measurements

Mice with a complete response to VB-85247 in the NMIBC model were rechallenged with MB49 tumor cells. Cells were resuspended in serum-free DMEM, and 100 μL containing 0.5 × 10^6^ cells were injected subcutaneously into the lower right flank. Tumor growth was monitored without further treatment. Tumors were measured using a caliper. Tumor size in mm^3^ was calculated using the formula V = (a × b^2^)/2, in which a and b are the long and short diameters of the tumor, respectively. Age-matched mice were used as controls.

### Flow cytometry

Single-cell suspensions from spleens, draining lymph nodes, and blood were resuspended in flow cytometry buffer containing PBS with 0.5 mmol/L EDTA and 2% FBS. The staining panels included markers for CD45 (BioLegend, 103116, RRID: AB_312981), CD3 (BioLegend, 100229, RRID: AB_11204249), CD11b (BioLegend, 101228, RRID: AB_893232), CD11c (BioLegend, 117348, RRID: AB_2563655), MHCII (BD Biosciences, 742894, RRID: AB_2734759), CD86 (BD Biosciences, 560582, RRID: AB_1727518), CD80 (BD Biosciences, 612773, RRID: AB_2870102), and CD49b (BioLegend, 103504, RRID: AB_313027). Prior to surface staining, cells were stained with surface dye Live/Dead Aqua (Life Technologies, L34966) followed by anti-CD16/32 to block Fc receptors (BD Biosciences, 553141, RRID: AB_394656). Blood samples were analyzed on the same day of staining. Spleen and lymph node samples were fixed overnight in 0.5% paraformaldehyde and analyzed on the next day. The data were acquired using Becton Dickinson LSRFortessa (BD Biosciences, RRID: SCR_018655) and analyzed with FlowJo software (BD Biosciences, RRID: SCR_008520).

### Statistical analysis

All statistical analysis was performed using Prism software (GraphPad, RRID: SCR_002798). Details of statistical analysis are found within each figure legend. A value of *P* < 0.05 was considered statistically significant.

### Data availability

Data for this study were generated at Genesis Biotechnology Group. All raw data supporting the findings of this study are available upon request from the corresponding author.

## Results

### STING agonist VB-85247 induced cellular gene expression

Activation of the STING signaling pathway by cyclic dinucleotides leads to the production of type I IFNs and proinflammatory cytokines in multiple cell types (10, 16). Consistent with this idea, VB-85247 induced target gene expression in human THP1 and mouse RAW 246.7 cell lines. The published synthetic STING agonist ADU-S100, known to induce antitumor immune responses, was utilized as a positive control (27). Stimulation of both human monocyte cell line THP1-Dual and mouse macrophagic cell line RAW-Dual with VB-85247 instigated elevated STING pathway target gene induction at 4.5 or 5 hour time points examined. Type I IFNs as well as IFN stimulatory genes such as IFIT1 and the proinflammatory cytokine IL6 were significantly upregulated upon stimulation with VB-85247 (Tables 1 and 2). VB-85247 consistently induced moderately higher levels of gene expression than an equal concentration of ADU-S100.

**Table 1.** qRT-PCR analysis of STING pathway target gene induction in cells stimulated with STING agonist VB-85247. STING pathway target gene induction in THP1-Duals cells.

THP1-Dual cells stimulated with VB-85247 at 10 μmol/L for 4.5 hours			
Gene	Control	ADU-S100	VB-85247
IFNβ	1	1,736	3,806
IFIT1	1	2,221	3,125
IL6	1	273	560

**Table 2.** qRT-PCR analysis of STING pathway target gene induction in cells stimulated with STING agonist VB-85247. STING pathway target gene induction in RAW-Dual cells.

RAW 246.7 cells stimulated with VB-85247 at 10 μmol/L for 5 hours			
Gene	Control	ADU-S100	VB-85247
IFNβ	1	1,736	3,806
IFIT1	1	2,221	3,125
IL6	1	273	560

### VB-85247 induces robust IFN responses in human primary cells

**Figure 1.**
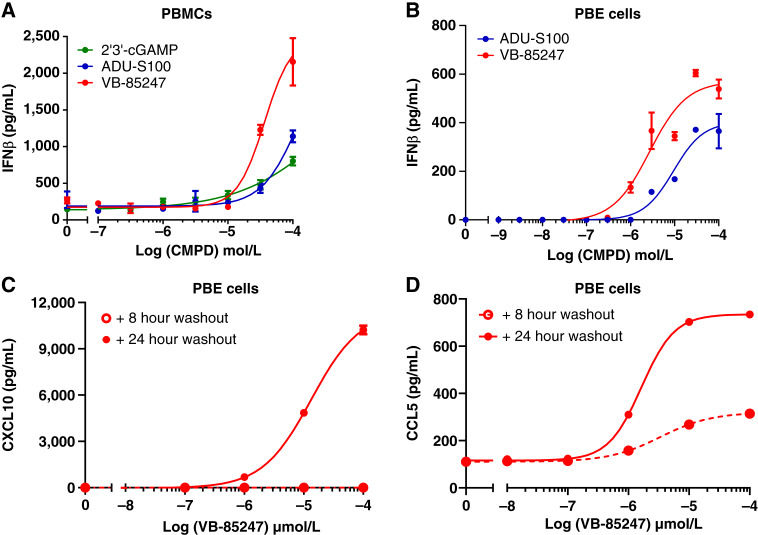
STING pathway target gene induction in human primary cells. **A,** IFNβ secretion from human PBMCs incubated with the indicated concentrations of VB-85247 for 2 hours. **B,** As in **A**, but levels of IFNβ secretion were measured from human PBE cells. **C,** Levels of CXCL10 secreted from human PBE cells incubated with indicated concentrations of VB-85247 for 2 hours, followed by incubation in fresh media for additional 6 or 22 hours. **D,** As in **C**, but the levels of CCL5 were measured.

We investigated the duration of exposure required to induce cytokine production to determine how long tumors would need to be exposed to VB-85247 to induce robust responses (Supplementary Fig. S2). The activity of VB-85247 after 30, 60, 90, and 120 minutes of exposure of human peripheral blood mononuclear cells (PBMC) was assessed by measuring STING downstream cytokines, including IFNα, IFNβ, CXCL10, TNFα, and IL6. The 30 minute exposure time resulted in minimal cytokine production compared with other exposure times, whereas the 90 and 120 minute exposure times resulted in robust stimulation of cytokine production in close temporal association with exposure time. Based on these results, the potency of VB-85247 was further investigated on primary human PBMCs and primary bladder epithelial (PBE) cells stimulated for 120 minutes. The natural STING ligand 2′, 3′-cGAMP and the reference compound ADU-S100 were used as controls. Notably, IFNβ was strongly upregulated in response to VB-85247 as compared with cGAMP and ADU-S100 in both human PBMCs and PBE cells (Fig. 1A and B). The IFNβ downstream chemokines CCL5 and CXCL10 are important for the recruitment of immune cells into the tumor microenvironment, driving the antitumor immune response (16, 17). Thus, we next assessed the levels of CCL5 and CXCL10 secreted by human PBE cells treated for 2 hours with VB-85247 followed by fresh media without compound for additional 6 hours (total 8 hours) or 22 hours (total 24 hours). The VB-85247 treatment significantly increased the production of CXCL10 and CCL5 at the 24 hour time point (Fig. 1C and D). Taken together, these results indicate that VB-85247 is biologically active within a 2 hour duration of exposure on both human bladder cells and PBMCs to induce robust cytokine responses.

In this experiment (Fig. 1) the PBMCs were homozygous for wild-type (WT) STING. There are four common human STING variants in addition to WT, known as HAQ, R232H, AQ, and R293Q. The variants are reported to occur with frequencies of 1.5% to 57.9% (28–30). We therefore evaluated VB-85247 response across donors with a range of STING variants. VB-85247 induced type I IFN responses in the PBMCs from all 12 human donors examined, including two WT/HAQ heterozygous donors, one HAQ/R232H heterozygous donor, one WT/R232H donor, and one R232H homozygous donor (Supplementary Fig. S3). THP1 cells express the HAQ variant and displayed a strong response to VB-85247 (Table 1). These data show that VB-85247 can act on normal human cells expressing a variety of STING SNP.

### Pharmacodynamic analysis of VB-85247 in a preclinical mouse model of bladder cancer

**Figure 2.**
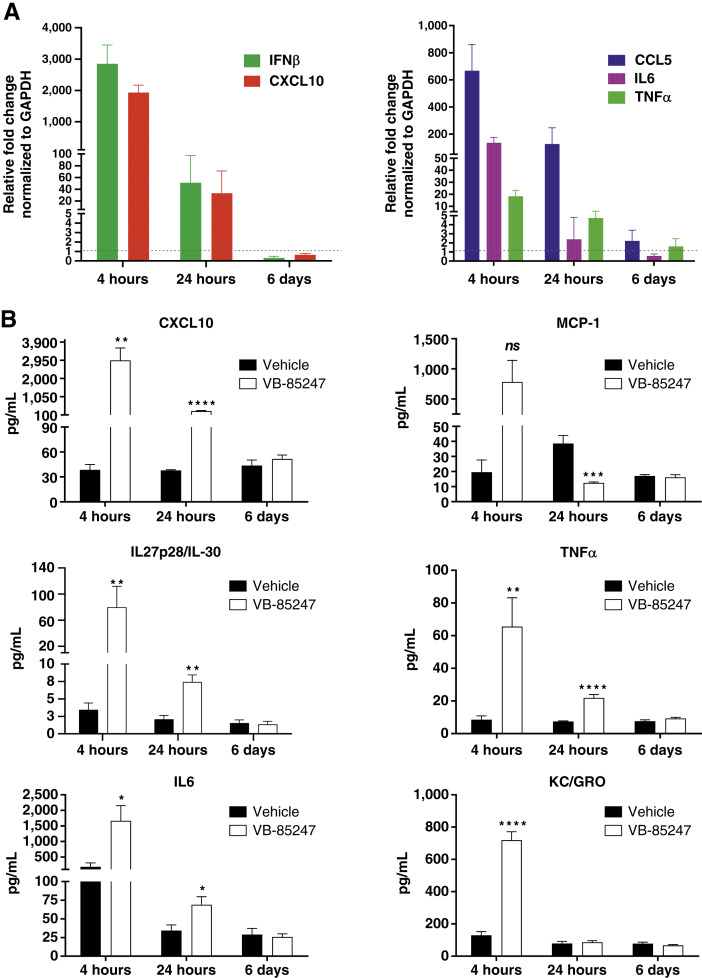
VB-85247 induces expression of immune cell–stimulating cytokines and chemokines *in vivo*. Mice bearing bladder tumors received a single-dose intravesical injection of vehicle or 40 μg (2 mg/mL) VB-85247. Bladder and serum samples were collected at 4 hours, 6 hours, and 6 days. **A,** Relative fold change in gene expression in bladders of tumor-bearing mice by RT-PCR. Data were normalized to corresponding GAPDH, and fold changes in gene induction were calculated by the comparative C_t_ method. **B,** Multiplex analysis of cytokines and chemokines in serum. Statistics by the unpaired Student test (two-tailed) for each indicated time point; significances are noted as *, *P* < 0.05; **, *P* < 0.01; ***, *P* < 0.001; ****, *P* < 0.0001; ns, not significant.

Next, we tested the biological effect of VB-85247 on STING pathway target gene induction in an experimental syngeneic mouse model of NMIBC using orthotopic implantation of MB49-luc bladder tumor cells into the bladders of the mice. Four days after tumor cell implantation, the animals were separated into treatment groups based on BLI signal with comparable mean tumor sizes for each group (Fig. 2A, day 4). The animals were treated with a single dose of 40 μg (2 mg/kg) VB-85247 or vehicle for 2 hours given via intravesical delivery. The 2 hour exposure time was selected to achieve high level of STING target gene expression on bladder urothelial cells consistent with the duration of *in vitro* exposure described in Fig. 1. STING pathway target gene induction was assessed locally in the whole bladder containing tumor by qRT-PCR analysis of mRNA and systemically by cytokine protein measurement in serum at 4 hours, 24 hours, and 6 days. Both the mRNA and cytokine analyses showed robust STING pathway activation and target gene induction compared with vehicle-treated animals. Particularly, intravesical instillation of VB-85247 induced elevated gene expression in the tumor-bearing bladders for IFNβ, CXCL10, CCL5, IL6, and TNFα at 4 hours after treatment, with the fold-induction of gene expression in the order of IFNβ > CXCL10 > CCL5 > IL6 > TNFα (Fig. 2A). Elevated levels were maintained at least up to 24 hours. The basal levels of gene expression were restored when examined on day 6 (Fig. 2A). The chemokine CXCL10 and proinflammatory cytokines TNFα and IL6 were also upregulated in the serum at 4 hours along with MCP-1, KC/GRO, and the antigen-presenting cell cytokine IL27. The significant differences were maintained at 24 hours, consistent with the gene expression (Fig. 2B). The serum cytokine levels returned to levels comparable with the control group by 6 days following treatment. Collectively, these results showed that intravesical instillation of VB-85247 elicits strong activation of STING signaling and target gene induction *in vivo*.

### Intravesical VB-85247 monotherapy provides durable robust efficacy and immunologic memory in the NMIBC model

**Figure 3.**
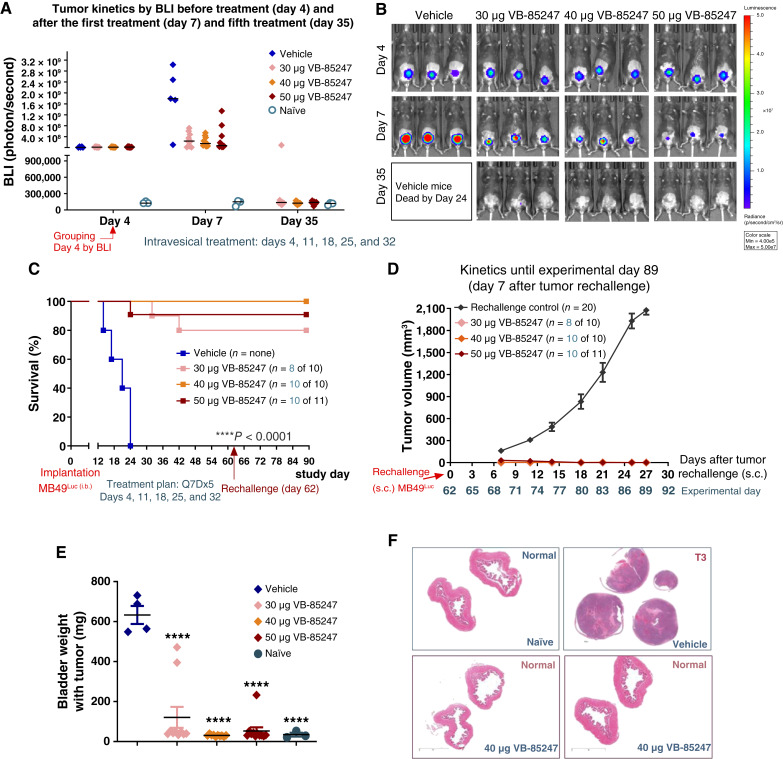
VB-85247 provides robust efficacy in a mouse model of NMIBC with lasting protection. Mice with established bladder tumors received vehicle or VB-85247 via intravesical instillation once per week for total of five doses (Q7Dx5): days 4, 11, 18, 25, and 32. **A,** The scatter blot shows the tumor growth over time, before the first dose on day 4, after the first dose on day 7, and after the last fifth dose on day 35. Individual mice are shown. Black bars, medians. **B,** Bioluminescent images of representative mice from each treatment group on days 4, 7, and 35. **C,** Kaplan–Maier survival curves for the vehicle control and VB-85247–treated groups. Survival is noted in parenthesis. Statistical significance was determined. **D,** Tumor kinetics of subcutaneously reimplanted MB49 cells on tumor-free mice initially treated with VB-8524. The number of tumor-free mice that were rechallenged is noted in the parentheses. Age-matched naïve mice were used as control. Mice with tumors 2,000 mm^3^ were euthanized. **E,** Bladder weight with the tumor in individual mice as outlined in **C**, or treated groups at study termination on day 89. Statistical significance was determined relevant to the vehicle group with one-way ANOVA, followed by the Tukey test. ****, *P* < 0.0001. **F,** Representative images of hematoxylin and eosin–stained bladder sections from the naïve control group and the 40 μg VB-85247 group with pathologic evaluation.

Next, we set out to evaluate the antitumor effect of VB-85247 following intravesical delivery in our syngeneic orthotopic murine NMIBC model. Because 2 hour exposure of the bladder urothelium to VB-85247 was optimal for STING target gene expression and cytokine secretion, in the efficacy arm, the orthotopic tumors were treated for 2 hours with VB-85247 at three different dose levels (30, 40, and 50 μg) or vehicle control. The animals received five weekly doses on days 4, 11, 18, 25, and 32. This weekly regimen was selected to provide acute stimulation followed by return to basal levels while avoiding chronic STING activation that can lead to toxicity or immunosuppression (23). Notably, as evident by the BLI signal, treatment with VB-85247 alone showed a significant regression of tumor growth within 3 days after the first dose at all tested dose levels, as compared with the control vehicle group, on day 7 (Fig. 3A and B). The 40 μg (2 mg/kg) dose seemed to be the lowest most efficacious dose, resulting in 100% complete responses by day 35 (Fig. 3A and B). Survival rates were 80% to 90% in the 30 μg (1.5 mg/kg) and 50 μg (2.5 mg/kg) VB-85247 groups and 100% in the 40 μg VB-85247 group. The median survival of the vehicle group was 21 days (Fig. 3C).

To examine whether intravesical monotherapy with VB-85247 induces immunologic memory, all animals that achieved complete responses were rechallenged with a s.c. inoculation of MB49-luc cells in parallel with age-matched controls on day 62. The mice received no further treatment, and the s.c. tumor growth was monitored. All of the rechallenged animals that had previously demonstrated complete responses rejected the tumor cells in contrast to the matched controls and displayed no mortality (Fig. 3D). At the end of the study, the assessments of complete responses to the primary bladder tumor were confirmed by the weight of bladder and histology relative to the control naïve nontumor-bearing mice (Fig. 3E and F). The lack of bladder tumors was also examined by the BLI signal via IVIS imaging (Supplementary Fig. S4). These results demonstrate that VB-85247 has the potential to induce a long-lasting antitumor response through activation of immune memory.

### STING agonist VB-85247 induced DC responses following intravesical administration

**Figure 4.**
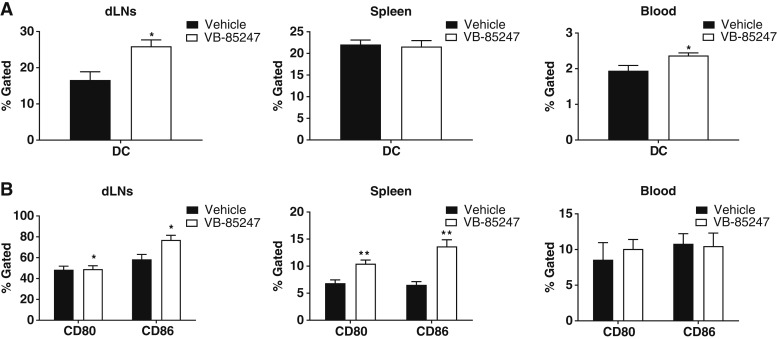
Single treatment of VB-85247 in the MB49-luc NMIBC model resulted in DC maturation and migration. Tumor-bearing mice received a single dose of VB-85247 via intravesical instillation. After 24 hours, draining lymph nodes (dLN), spleen, and blood were harvested and stained for flow cytometry. Bar plots show DCs (CD11c^+^MHCII^+^) as percent of CD45^+^CD11b^+^ cells (**A**) and costimulatory CD80 and CD86 molecules as percent of total DCs (**B**). Statistical significance was calculated using the unpaired *t* test. Error bars, ± SEM. *, *P* < 0.05; **, *P* < 0.01.

Numerous studies have shown that the STING pathway plays an important role in the regulation of DC activation and maturation in cancer (16, 31, 32). Immature DCs collect antigens throughout the body but live only a short time unless they get activated in the context of appropriate maturation signals, including cytokines that can be induced by STING activation. Activated DCs upregulate expression of CD80 (B7-1) and CD86 (B7-2), which provide costimulation to T cells for activation. Mature DCs migrate to lymphoid organs such as lymph nodes and the spleen, and the cells then can live for several weeks, presenting antigen to T cells (15, 33). We therefore examined the maturation/activation phenotype of DCs 24 hours after a single intravesical dose of 40 μg (2 mg/kg) of VB-85247 in mice bearing bladder tumors. VB-85247 induced mobilization of DCs (CD11c^+^MHCII^+^) in the blood and draining lymph nodes (Fig. 4A). In addition, there was a significant increase in the proportions of activated DCs, as measured by CD80 and CD86 expression in the draining lymph nodes and spleen within 24 hours when compared with their counterparts in the control vehicle group (Fig. 4B). These results are consistent with the previously observed *in vivo* cytokine and chemokine profiles (Fig. 2). Taken together, these results demonstrate that local administration of VB-85247 in the bladder can induce systemic immune responses in the mouse tumor model.

### VB-85247 is markedly superior to BCG treatment in the mouse model of NMIBC

**Figure 5.**
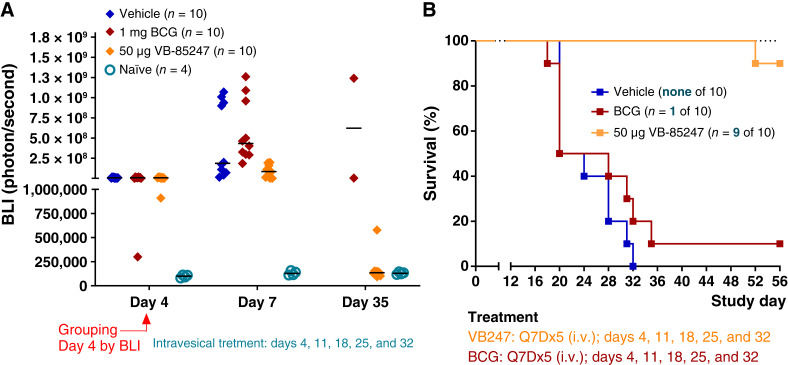
VB-85247 is markedly superior to BCG treatment in the mouse model of NMIBC. Mice bearing bladder tumors were treated with 1 mg BCG or 50 μg VB-85247 via intravesical delivery once per week for total of five doses (Q7Dx5): days 4, 11, 18, 25, and 32. **A,** Tumor growth kinetics of MB49-luc cells based on BLI signal on days 4, 7, and 35. Individual mice are shown. Black bars, medians. Statistical significance was determined relevant to the control vehicle group with one-way ANOVA, followed by the Tukey test on day 7, **, *P* < 0.0039. **B,** Kaplan–Maier survival curves for the vehicle control and VB-85247 treatment groups. The surviving animals at the end of the study are noted from the number of animals at the start of the experiment.

About 40% of patients with NMIBC fail intravesical BCG treatment (4–6). These patients are in urgent need of alternative treatment options. BCG efficacy against MB49 orthotopic bladder tumors in mice is unclear given divergent literature reports (34–39). We therefore compared BCG therapy to VB-85247 therapy using the NMIBC MB49-luc mouse model. BCG was given at 1 mg per mouse, as previously described (38), and VB-85247 at 50 μg. A single dose of BCG induced gene expression in the bladders of tumor-bearing mice, as shown in Supplementary Fig. S5, confirming that the BCG was biologically active. However, in the efficacy model of five weekly doses, by day 35, the only two surviving mice in the BCG-treated mice retained tumors, whereas 9 of 10 VB-85247 displayed low BLI signals equal to the naïve group that was not instilled with MB49-luc cells, demonstrating robust control of the original tumors. Three days after the first treatment (day 7), the 50 μg VB-85247 group showed suppression of tumor growth relative to the vehicle controls or BCG-treated groups, confirming a rapid onset of antitumor activity by VB-87247 (Fig. 5A). Overall, BCG was not effective in this model as only 1 of 10 mice survived 56 days following tumor instillation into the bladder (Fig. 5B). However, treatment with 50 μg VB-85247 resulted in the survival of 9 of 10 mice until the study was terminated on day 56, whereas all mice in the vehicle group were euthanized by day 32 due to body weight loss. The difference in survival was significant in the Mantel–Cox log-rank test (*P* < 0.0001); the median survival was 22 days for the vehicle control, 24 days for the BCG group, and undefined (>56 days) for the VB-85247–treated group. Thus, VB-85247 provides potent and durable remission of tumors in this BCG-unresponsive MB49 model of NMIBC.

### Combination immunotherapy of VB8-85247 with PD**-**1 blockade

**Figure 6.**
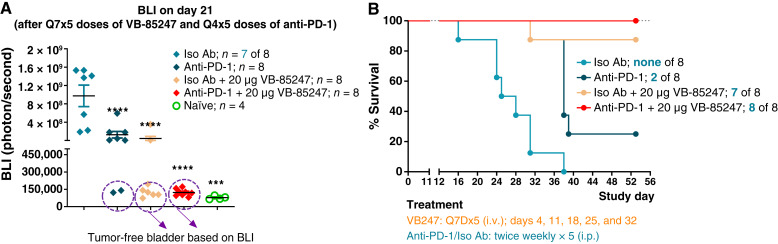
VB-85247 provides an additive effect to anti-PD-1 immunotherapy in the mouse model of NMIBC. Mice bearing bladder tumors were treated on days 4, 11, 18, 25, and 32 with 20 μg VB-85247 via intravesical delivery in combination with anti-PD-1 (10 mg/kg) or equal amount of isotype control twice weekly for a total of five doses. Control groups were treated with isotype antibody or anti-PD-1 (10 mg/kg) alone. **A,** Bioluminescent signal from bladder tumors on day 21 after tumor instillation. Individual mice are shown. Black bars, medians. Statistical significance was determined relevant to control isotype group with one-way ANOVA, followed by the Tukey test. ****, *P* < 0.0001. **B,** Kaplan–Maier survival curves. The surviving animals at the end of the study and the initial number of animals enrolled in the study are shown. Statistical significance, ***, *P* < 0.0001 (Mantel–Cox test). Iso Ab, isoflurane antibody.

Pembrolizumab, an anti-PD-1 immuno-oncology agent, is an alternative choice for patients who fail BCG treatment. During the KEYNOTE-057 trial, patients who failed BCG therapy received pembrolizumab 200 mg every 3 weeks and showed efficacy with 40% of these patients achieving complete response (8). Addition of anti-PD-1 to STING therapy has been reported to increase efficacy in preclinical tumor models (40). We therefore compared the efficacy of VB-85247 to anti-PD-1 monotherapy and investigated the potential benefit of VB-85247 in combination with anti-PD-1 in our orthotopic NMIBC MB49-luc mouse model. Anti-PD-1 antibody or its isotype control was given at 10 mg/kg by i.p. injection twice weekly for a total of five doses. VB-85247 was dosed by the intravesical route at 20 μg with isotype control or in combination with anti-PD-1 for a total of 6 weekly administrations. By day 21 all eight mice treated with the combination of anti-PD-1 plus 20 μg VB-85247 displayed robust tumor control, whereas only six of eight mice receiving 20 μg VB-85247 with the isotype antibody displayed similar efficacy, suggesting the potential of combination therapy for more rapid efficacy. By contrast, only two of eight mice treated with anti-PD-1 alone displayed robust tumor control (Fig. 6A). By the end of the study, only two of eight mice treated with anti-PD-1 alone achieved complete responses (Fig. 6B), indicating that the MB49 orthotopic model may be considered a model of anti-PD-1–resistant NMIBC. In the end, the combination of anti-PD-1 with 20 μg VB-85247 resulted in 100% complete response compared with 87.5% for 20 μg VB-85247 with the isotype control.

## Discussion

Adjuvant intravesical instillation with various chemotherapy agents and BCG are currently treatment strategies for patients with NMIBC, but many patients either fail initial therapy or develop recurrent tumors within a few years (4, 6). There is therefore a major unmet medical need for more effective therapies as the only other standard-of-care alternative is radical cystectomy, which many subjects may choose not to undergo due to significant morbidity, mortality, and impact on the quality of life (5). Toward this goal, our group has developed a novel STING agonist, VB-85247. A key mechanism of action of the STING agonist for induction of antitumor immune responses is the production of type I IFN, particularly IFNβ. In this study, we show that VB-85247 is active on PBMCs from human subjects with a range of STING polymorphisms and strongly induces IFNβ production by human PBMCs and PBE cells. Moreover, VB-85247 was more potent than the reference compound ADU-S100 (Fig. 1A and B). VB-85247 also induced the IFN-dependent chemokines CXCL10 (IP-10) and CCL5, which are known to be important for the recruitment of immune effector cells into the tumor microenvironment. In alignment with these *in vitro* data, instillation of single-dose 40 μg (2 mg/kg) VB-85247 *in situ* induced rapid gene expression of key cytokine genes (IFNβ, CXCL10, CCL5, IL6, and TNFα) in bladders of mice bearing NMIBC tumors (Fig. 2A). Most importantly, local intravesical VB-85247 single-dose treatment elicited a systemic immune response as evident by increases in serum cytokines (Fig. 2B) and DC mobilization and activation in the blood, draining lymph nodes, and spleen within 24 hours (Fig. 4). These findings identify the *in vivo* mechanisms of action for the potential utility of VB-85247 in cancer immunotherapy through activation of the STING signaling pathway.

In the efficacy studies with the NMIBC mouse model, monotherapy with VB-85247 via intravesical delivery results in tumor regression starting after the first treatment, achieving up to 100% complete response rate at the 40 μg (2 mg/kg) dose level after five weekly treatments. The treatment was well tolerated, eliciting a strong and durable antitumor immune response without any mortality, and following eradication of the tumors, the bladders displayed normal histopathology. Moreover, the mice cured of their primary bladder tumors rejected fresh inoculations of tumor cells with no further treatment, demonstrating an induction of immunologic memory in all VB-85247–treated animals.

Gene expression analysis of VB-85247–treated tumors revealed upregulation of CXCL10 and CCL5 chemokines, essential for T-cell trafficking. These results align with the classic notion that type I IFNs upregulate costimulatory and MHC class molecules on DCs and promotes DC antigen cross-priming to CD8^+^ T cells. In addition, it has been shown that type I IFN–dependent DCs promote NK cell effector function as well (41–43). Because activation of STING signaling can amplify both T- and NK cell–mediated antitumor response, in future studies, we will address the contribution of these immune cells to VB-85247 therapy. It will be also interesting to explore whether VB-85247 would amplify different cell responses based on the tumor microenvironment.

When tested in our NMIBC model, and with the same treatment schedule, we found VB-85247 to be superior to intravesical BCG or systemic anti-PD-1 therapy in terms of tumor control and survival, indicating the potential utility of VB-85247 in the treatment of BCG or anti-PD-1–unresponsive patients. Although our data suggest that VB-85247 may have the potential to improve anti-PD-1 responses in patients with NMIBC, detailed dose–response studies in our NMIBC mouse model will be needed to fully understand the potential benefit. Another important future area of investigation will be to determine whether VB-85247 can provide combination benefit with other approved checkpoint blockade agents such as anti-CTLA4 and anti-LAG3.

To date, clinical studies of STING agonists administered by i.t. injection have resulted in only limited efficacy as monotherapy or in combination with anti-PD-1 (22, 24, 26). This lack of efficacy may be due at least in part to the very rapid clearance of STING agonists from human tumors following i.t. injection. For example, ADU-S100 was reported to reach maximum concentrations in systemic circulation by 15 minutes, the first time point examined after injection into tumors of patients (26), and the STING agonist E7766 was tested in an animal model of NMIBC via intravesical delivery for 45 minutes (44) but not reported in clinical studies for NMIBC. We determined that 2 hour exposure of human cells, including bladder epithelial cells, to VB-85247 results in robust cytokine responses, whereas shorter times such as 30 minutes gave only minimal responses (Fig. 1). The 2 hour intravesical exposure utilized in our mouse model of NMIBC and in clinical practice for BCG therapy therefore provides a sustained duration of exposure sufficient for robust STING activation *in vitro* and *in vivo*. Importantly, intravesical treatment can provide stimulation of STING not only in tumor cells but also in the normal cells of the bladder to induce beneficial cytokine production to stimulate antitumor immune responses. It is also important to note that local administration of VB-85247 via intravesical delivery has the potential to limit systemic exposure and thus any systemic toxicity. Based on these data, we propose that STING agonist VB-85247 administered by the intravesical route has the potential to improve treatment of patients with bladder cancer and extend the clinical benefit of anti-PD-1. VB-85247 is being advanced into clinical development for the treatment of NMIBC.

## Supplementary Material

Table S1 supplementary

Figure S1 supplementary figure 1

Figure S2 supplementary figure 2

Figure S3 supplementary figure 3

Figure S4 supplementary figure 4

Figure S5 supplementary figure 5
