# Supplementary material for: STING Agonist VB-85247 Induces Durable Antitumor Immune Responses by Intravesical Administration in a Non–Muscle-Invasive Bladder Cancer
Source: Cancer Res. 2024 Dec 19;85(7):1287–96. doi: 10.1158/0008-5472.CAN-24-1022 (PMC11966111; doi:10.1158/0008-5472.CAN-24-1022)
Supplement: Table S1 — supplementary [file can-24-1022_table_s1_suppst1.pptx]

## Slide 1
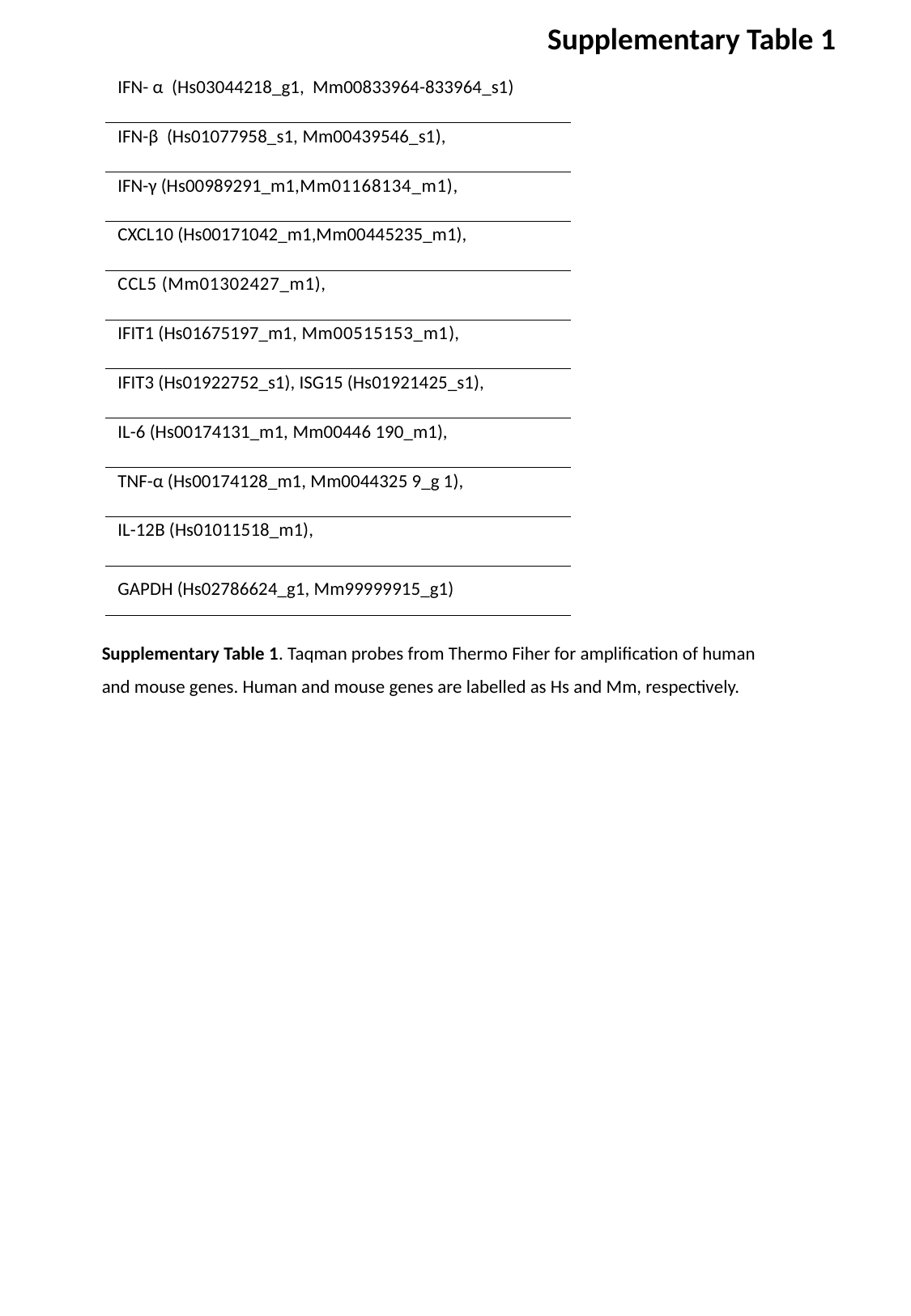

Supplementary Table 1
| IFN- α (Hs03044218\_g1, Mm00833964-833964\_s1) |
| --- |
| IFN-β (Hs01077958\_s1, Mm00439546\_s1), |
| IFN-γ (Hs00989291\_m1,Mm01168134\_m1), |
| CXCL10 (Hs00171042\_m1,Mm00445235\_m1), |
| CCL5 (Mm01302427\_m1), |
| IFIT1 (Hs01675197\_m1, Mm00515153\_m1), |
| IFIT3 (Hs01922752\_s1), ISG15 (Hs01921425\_s1), |
| IL-6 (Hs00174131\_m1, Mm00446 190\_m1), |
| TNF-α (Hs00174128\_m1, Mm0044325 9\_g 1), |
| IL-12B (Hs01011518\_m1), |
| GAPDH (Hs02786624\_g1, Mm99999915\_g1) |
Supplementary Table 1. Taqman probes from Thermo Fiher for amplification of human and mouse genes. Human and mouse genes are labelled as Hs and Mm, respectively.
