# Supplementary material for: STING Agonist VB-85247 Induces Durable Antitumor Immune Responses by Intravesical Administration in a Non–Muscle-Invasive Bladder Cancer
Source: Cancer Res. 2024 Dec 19;85(7):1287–96. doi: 10.1158/0008-5472.CAN-24-1022 (PMC11966111; doi:10.1158/0008-5472.CAN-24-1022)
Supplement: Figure S1 — supplementary figure 1 [file can-24-1022_figure_s1_suppsf1.pptx]

## Slide 1
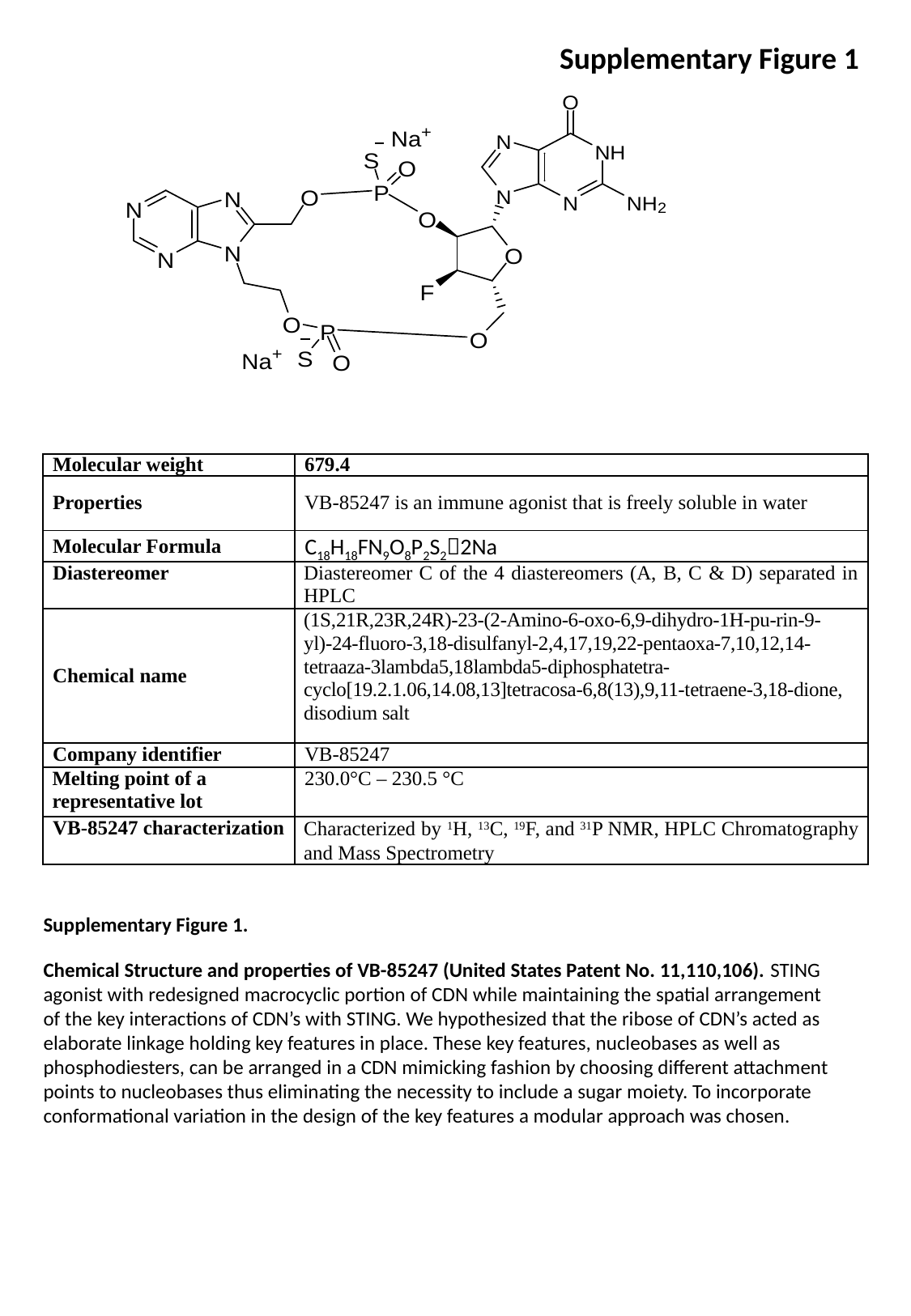

Supplementary Figure 1
| Molecular weight | 679.4 |
| --- | --- |
| Properties | VB-85247 is an immune agonist that is freely soluble in water |
| Molecular Formula | C18H18FN9O8P2S22Na |
| Diastereomer | Diastereomer C of the 4 diastereomers (A, B, C & D) separated in HPLC |
| Chemical name | (1S,21R,23R,24R)-23-(2-Amino-6-oxo-6,9-dihydro-1H-pu-rin-9-yl)-24-fluoro-3,18-disulfanyl-2,4,17,19,22-pentaoxa-7,10,12,14-tetraaza-3lambda5,18lambda5-diphosphatetra-cyclo[19.2.1.06,14.08,13]tetracosa-6,8(13),9,11-tetraene-3,18-dione, disodium salt |
| Company identifier | VB-85247 |
| Melting point of a representative lot | 230.0°C – 230.5 °C |
| VB-85247 characterization | Characterized by 1H, 13C, 19F, and 31P NMR, HPLC Chromatog­raphy and Mass Spectrometry |
Supplementary Figure 1.
Chemical Structure and properties of VB-85247 (United States Patent No. 11,110,106). STING agonist with redesigned macrocyclic portion of CDN while maintaining the spatial arrangement of the key interactions of CDN’s with STING. We hypothesized that the ribose of CDN’s acted as elaborate linkage holding key features in place. These key features, nucleobases as well as phosphodiesters, can be arranged in a CDN mimicking fashion by choosing different attachment points to nucleobases thus eliminating the necessity to include a sugar moiety. To incorporate conformational variation in the design of the key features a modular approach was chosen.
