# Supplementary material for: STING Agonist VB-85247 Induces Durable Antitumor Immune Responses by Intravesical Administration in a Non–Muscle-Invasive Bladder Cancer
Source: Cancer Res. 2024 Dec 19;85(7):1287–96. doi: 10.1158/0008-5472.CAN-24-1022 (PMC11966111; doi:10.1158/0008-5472.CAN-24-1022)
Supplement: Figure S2 — supplementary figure 2 [file can-24-1022_figure_s2_suppsf2.pptx]

## Slide 1
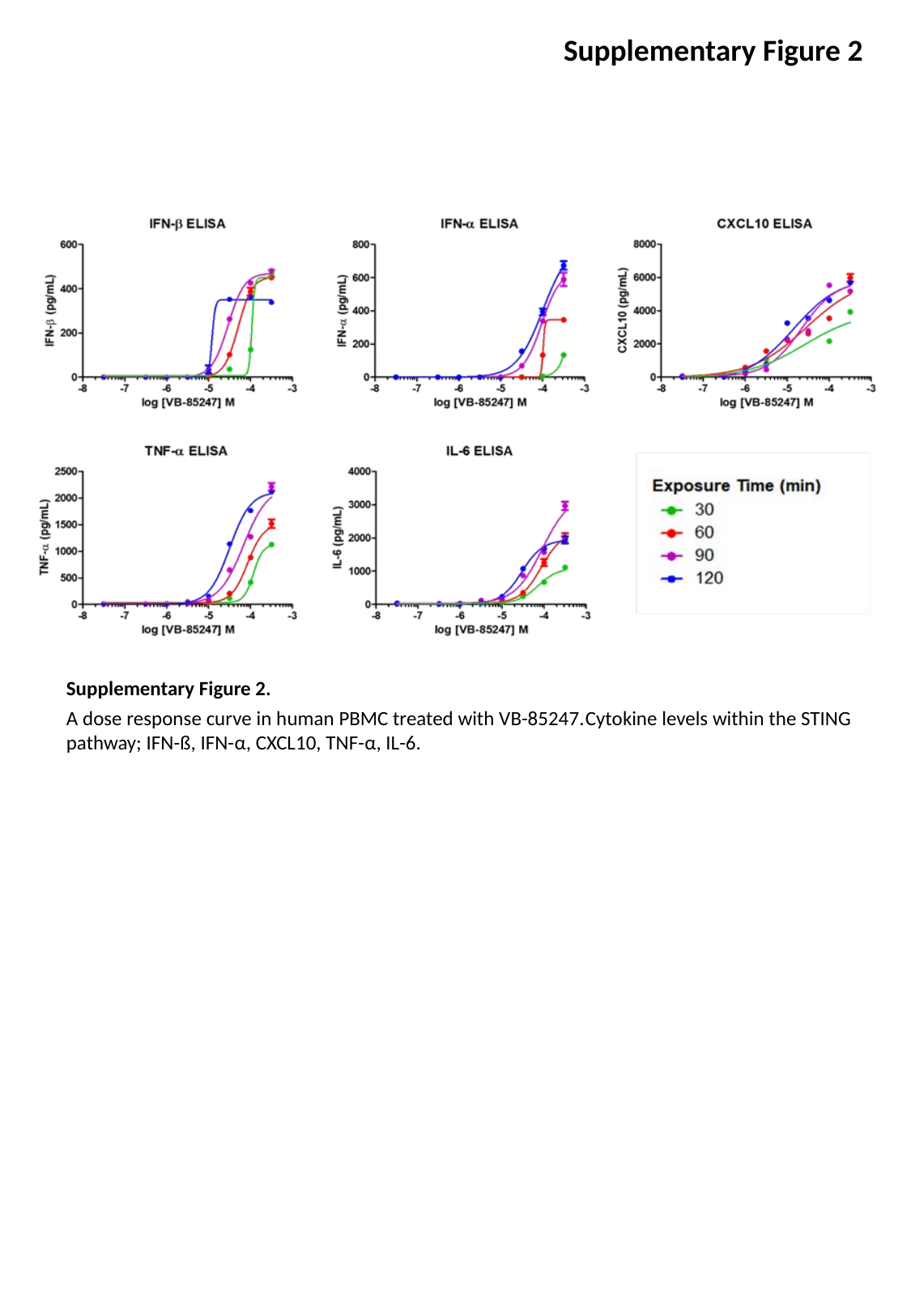

Supplementary Figure 2
Supplementary Figure 2.
A dose response curve in human PBMC treated with VB-85247.Cytokine levels within the STING pathway; IFN-ß, IFN-α, CXCL10, TNF-α, IL-6.
