# Supplementary material for: STING Agonist VB-85247 Induces Durable Antitumor Immune Responses by Intravesical Administration in a Non–Muscle-Invasive Bladder Cancer
Source: Cancer Res. 2024 Dec 19;85(7):1287–96. doi: 10.1158/0008-5472.CAN-24-1022 (PMC11966111; doi:10.1158/0008-5472.CAN-24-1022)
Supplement: Figure S3 — supplementary figure 3 [file can-24-1022_figure_s3_suppsf3.pptx]

## Slide 1
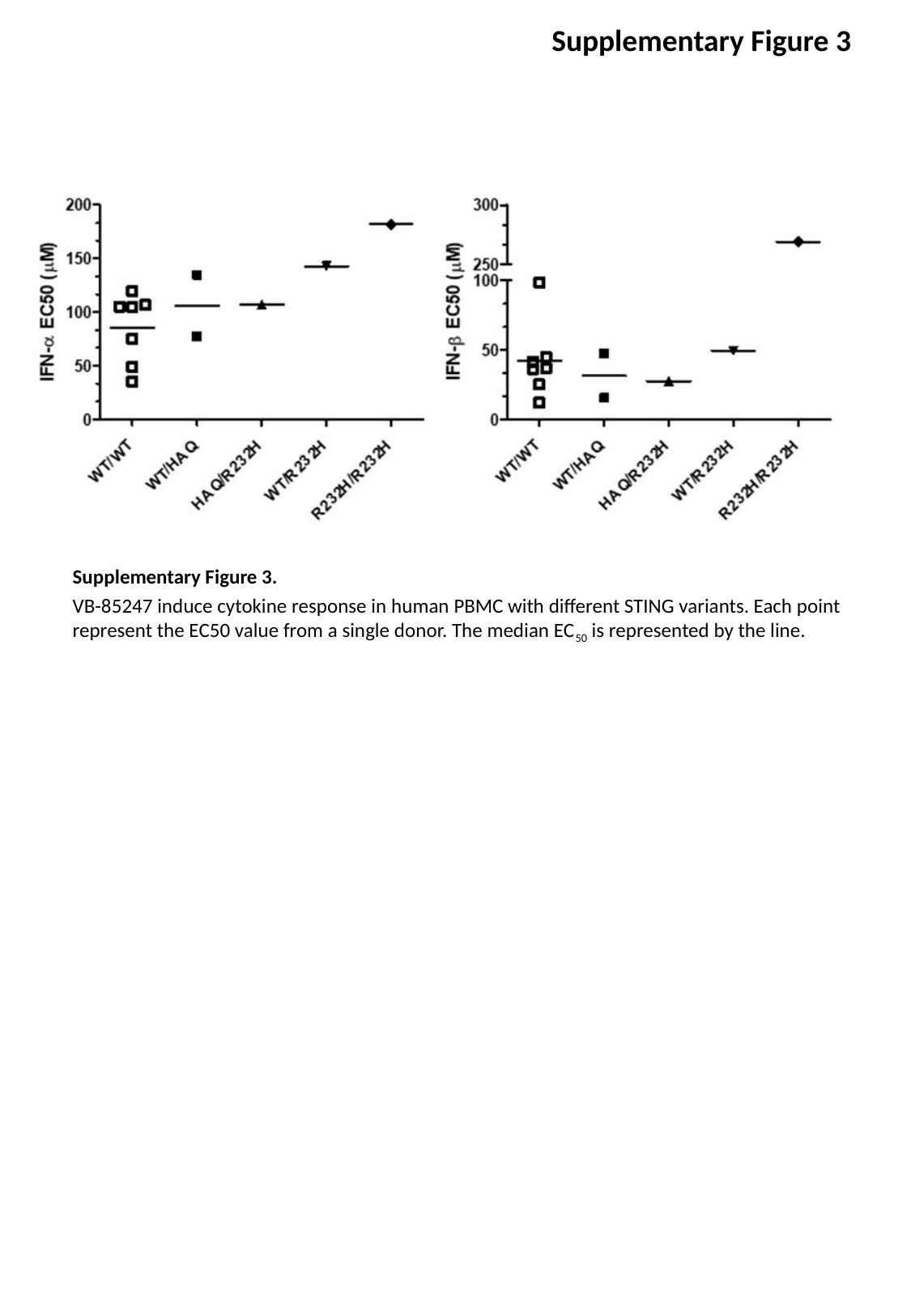

Supplementary Figure 3
Supplementary Figure 3.
VB-85247 induce cytokine response in human PBMC with different STING variants. Each point represent the EC50 value from a single donor. The median EC50 is represented by the line.
