# Supplementary material for: STING Agonist VB-85247 Induces Durable Antitumor Immune Responses by Intravesical Administration in a Non–Muscle-Invasive Bladder Cancer
Source: Cancer Res. 2024 Dec 19;85(7):1287–96. doi: 10.1158/0008-5472.CAN-24-1022 (PMC11966111; doi:10.1158/0008-5472.CAN-24-1022)
Supplement: Figure S4 — supplementary figure 4 [file can-24-1022_figure_s4_suppsf4.pptx]

## Slide 1
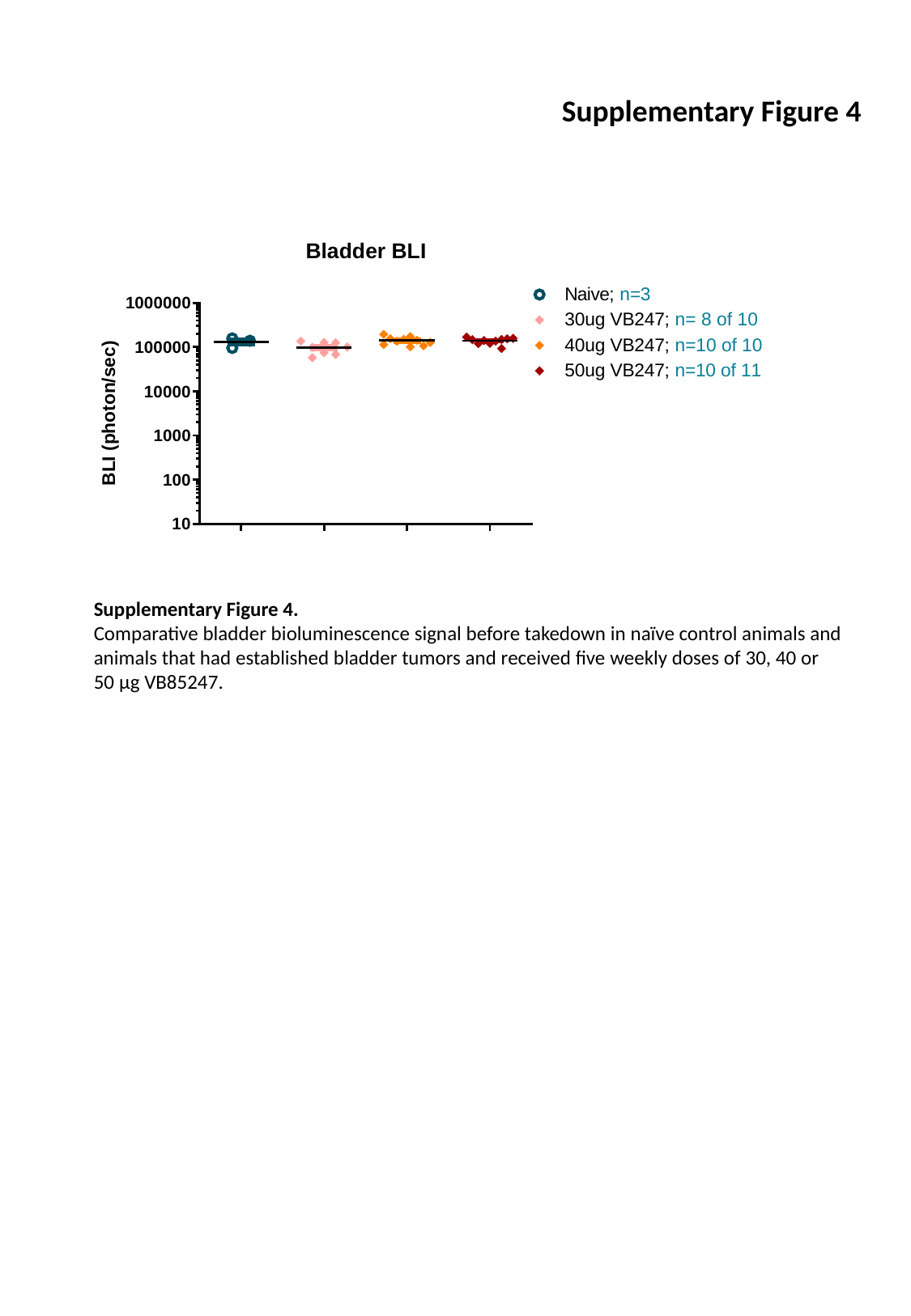

Supplementary Figure 4
Supplementary Figure 4.
Comparative bladder bioluminescence signal before takedown in naïve control animals and animals that had established bladder tumors and received five weekly doses of 30, 40 or 50 µg VB85247.
