# Supplementary material for: STING Agonist VB-85247 Induces Durable Antitumor Immune Responses by Intravesical Administration in a Non–Muscle-Invasive Bladder Cancer
Source: Cancer Res. 2024 Dec 19;85(7):1287–96. doi: 10.1158/0008-5472.CAN-24-1022 (PMC11966111; doi:10.1158/0008-5472.CAN-24-1022)
Supplement: Figure S5 — supplementary figure 5 [file can-24-1022_figure_s5_suppsf5.pptx]

## Slide 1
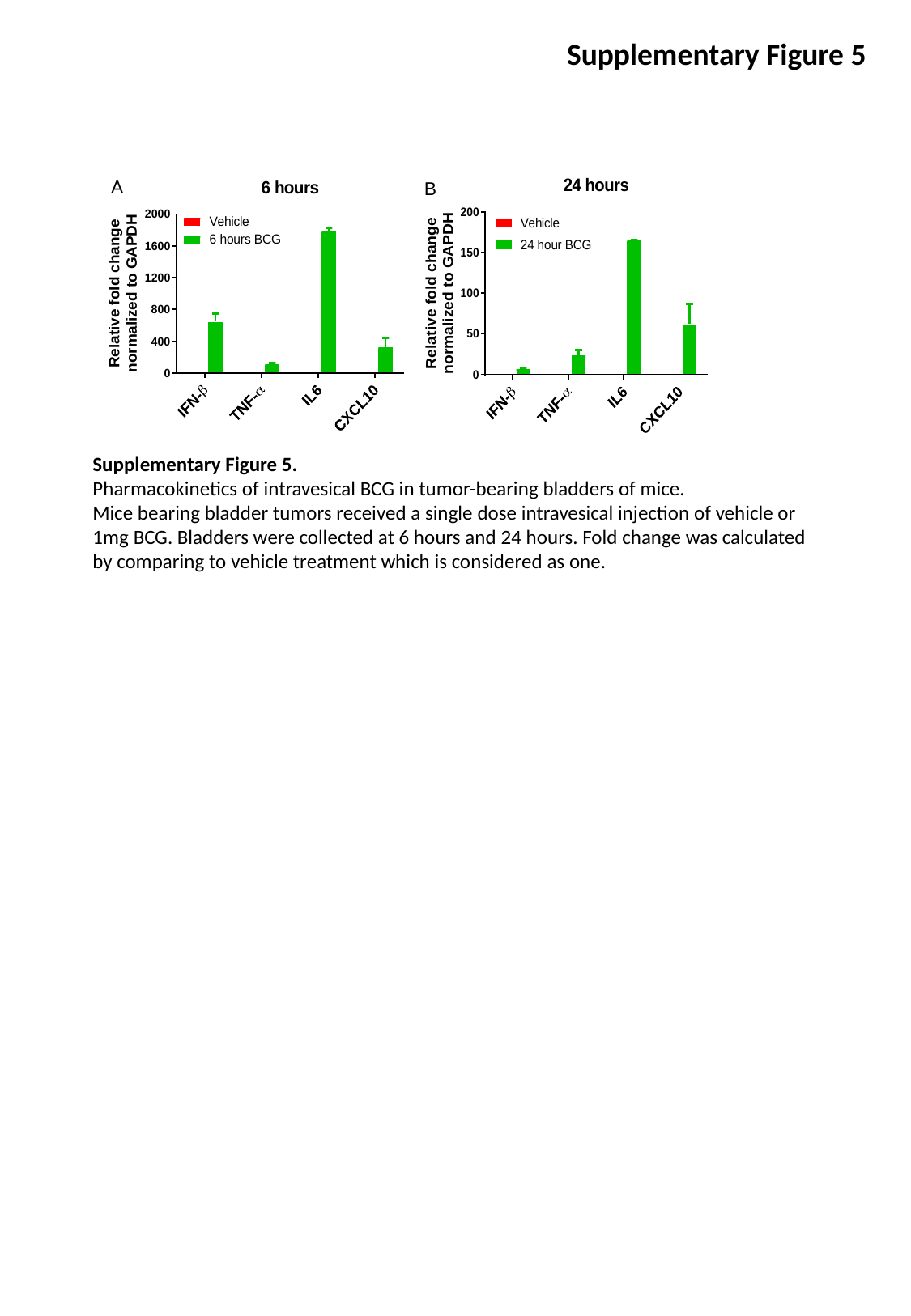

Supplementary Figure 5
A
B
Supplementary Figure 5.
Pharmacokinetics of intravesical BCG in tumor-bearing bladders of mice.
Mice bearing bladder tumors received a single dose intravesical injection of vehicle or 1mg BCG. Bladders were collected at 6 hours and 24 hours. Fold change was calculated by comparing to vehicle treatment which is considered as one.
